# Effect of the JAK2/STAT3 inhibitor SAR317461 on human glioblastoma tumorspheres

**DOI:** 10.1186/s12967-015-0627-5

**Published:** 2015-08-18

**Authors:** Rajesh Mukthavaram, Xiao Ouyang, Rohit Saklecha, Pengfei Jiang, Natsuko Nomura, Sandeep C Pingle, Fang Guo, Milan Makale, Santosh Kesari

**Affiliations:** Translational Neuro-Oncology Laboratories, Moores Cancer Center, UC San Diego, 3855 Health Sciences Drive, MC#0819, La Jolla, CA 92093-0819 USA; Department of Neurosciences, UC San Diego, La Jolla, CA USA; Department of Orthopedic Surgery, Xuzhou 3rd Hospital, Affiliated Hospital of Jiangsu University, No. 131 Huancheng Road, 221005 Xuzhou, China; Laboratory of Tumor Targeted Therapy, Shanghai Advanced Research Institute, Chinese Academy of Sciences, University of Chinese Academy of Sciences, 99 Haike Road, 201210 Shanghai, China; Department of Radiation Medicine and Applied Sciences, UC San Diego, La Jolla, CA USA

**Keywords:** Glioblastoma (GBM), JAK2, STAT3, Transcription factor, Tumorsphere, TCGA

## Abstract

**Background:**

The STAT3 transcription factor is a major intracellular signaling protein and is frequently dysregulated in the most common and lethal brain malignancy in adults, glioblastoma multiforme (GBM). Activation of STAT3 in GBM correlates with malignancy and poor prognosis. The phosphorylating signal transducer JAK2 activates STAT3 in response to cytokines and growth factors. Currently there are no JAK-STAT pathway inhibitors in clinical trials for GBM, so we sought to examine the anti-GBM activity of SAR317461 (Sanofi-Aventis), a newer generation, highly potent JAK2 inhibitor that exhibits low toxicity and good pharmacokinetics. SAR317461 was initially approved for patient testing in the treatment of primary myelofibrosis (PMF), and has shown activity in preclinical models of melanoma and pulmonary cancer, but has not been tested in GBM.

**Methods:**

We hypothesized that a potent small molecule JAK2 inhibitor could overcome the heterogeneous nature of GBM, and suppress a range of patient derived GBM tumorsphere lines and immortalized GBM cell lines. We treated with SAR317461 to determine IC_50_ values, and using Western blot analysis we asked whether the response was linked to STAT3 expression. Western blot analysis, FACS, and cell viability studies were used to identify the mechanism of SAR317461 induced cell death.

**Results:**

We report for the first time that the JAK2 inhibitor SAR317461 clearly inhibited STAT3 phosphorylation and had substantial activity against cells (IC_50_ 1–10 µM) from 6 of 7 different patient GSC derived GBM tumorsphere lines and three immortalized GBM lines. One patient GSC derived line did not constitutively express STAT3 and was more resistant to SAR317461 (IC_50_ ≈25 µM). In terms of mechanism we found cleaved PARP and clear apoptosis following SAR317461. SAR317461 also induced autophagy and the addition of an autophagy inhibitor markedly enhanced cell killing by SAR317461.

**Conclusions:**

We conclude that SAR317461 potently inhibits STAT3 phosphorylation and that it has significant activity against those GBM cells which express activated STAT3. Further studies are warranted in terms of the potential of SAR317461 as single and combined therapy for selectively treating human patients afflicted with GBMs expressing activation of the JAK2-STAT3 signaling axis.

## Background

**Fig. 1:**
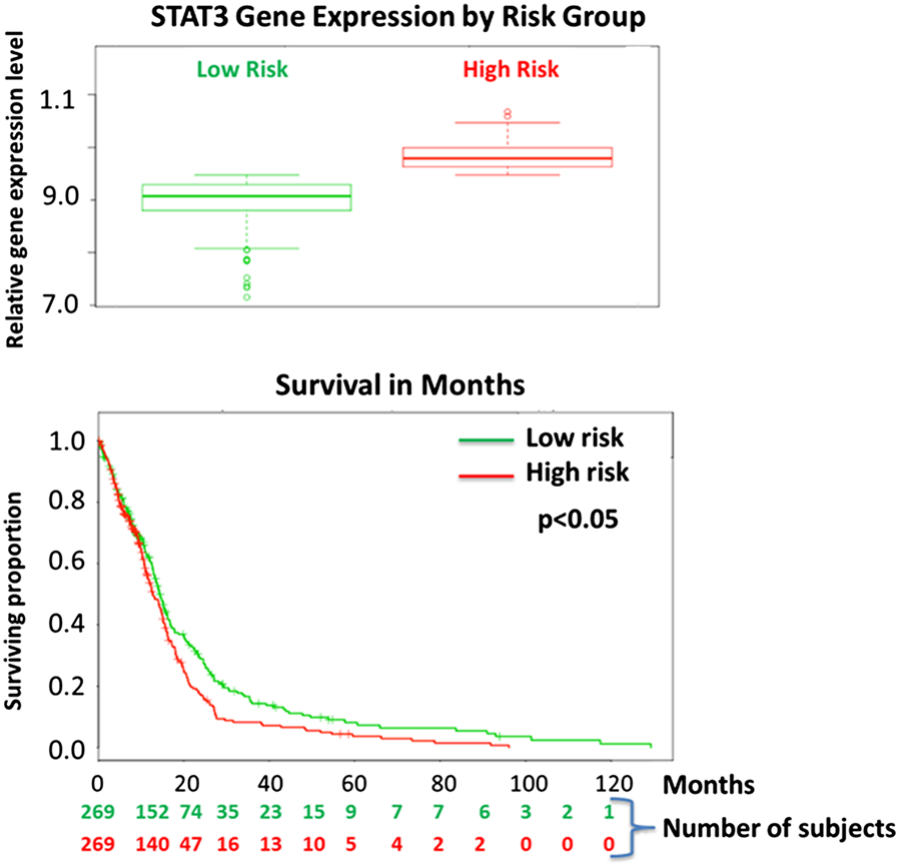
TCGA data show that STAT3 expression in GBM correlates with lower patient survival. **a** Relative STAT3 expression levels according to GBM patient risk group based on TCGA genetic profiling. Higher risk individuals had higher STAT3 expression and their expression levels are denoted by *red symbols*, while lower STAT3 expression that was associated with lower survival (**b**) and lower risk is shown in *green*. **b** Survival curves, high risk in *red* indicates reduced survival; the lower risk group survival is denoted by *green curve*. P values calculated using log rank indicate significance at the 95 % confidence level (p < 0.05). Survival analysis was censored by survival months which means even though patients may have been lost to follow-up, partial data was acquired and incorporated in the survival curves according to study month.

Glioblastoma (GBM) is the most common brain cancer in adults and is notorious for its diffuse invasion, heterogeneity, treatment resistance, and dismal 5 year patient survival of less than 5 % [1, 2]. The current front-line GBM therapeutic temozolomide (TMZ) combined with intensive megavoltage radiotherapy exerts some benefit, but falls far short in terms of efficacy and systemic toxicity [3, 4]. The growth of human GBM cell lines and their TMZ resistance has been inhibited by RNA based knockdown and by small molecule inhibition of STAT3, a latent transcription factor that is dysregulated in many cancers [5–10]. Constitutive activation of STAT3 in gliomas is positively associated with tumor grade [6, 7, 11], and TCGA data reveals that elevated STAT3 in GBM is associated with reduced patient survival (Fig. 1—see “Methods”).

STAT3 is a major intracellular signaling hub and its downstream targets include Bcl-2, Bcl-X_L_, c-myc, survivin, cyclin D1, vascular endothelial growth factor (VEGF), among others [12–15]. STAT3 is activated when the signal transduction protein janus kinase 2 (JAK2) is stimulated by any of a variety of cytokines (IL-6, IL-11) and growth factors (EGF, TGF-α, PDGF and HGF) to phosphorylate the STAT3 tyrosine 705 residue [13, 14]. Constitutively activated STAT3 promotes tumor cell cycle progression and survival, elicits angiogenesis, and suppresses the immune response to tumors [16]. STAT3 is reportedly not essential to the survival of normal cells, but is critical to many types of cancer cells, making it a potentially valuable therapeutic target [16].

While positive results have been reported for JAK-STAT inhibition in preclinical GBM models, no human glioma JAK-STAT inhibitor based clinical studies have yet been performed, although one trial for a small molecule JAK-STAT inhibitor, WP1066, is pending (U.S. National Clinical Trials Database—https://clinicaltrials.gov/) [8–10]. This relative lack of activity may be a result of two considerations, (1) the preclinical results with JAK-STAT inhibition in GBM are variable, and (2) STAT3 signaling is nuanced since STAT3 can act, paradoxically, as a tumor promoter or suppressor, depending on factors that require further clarification [17, 18]. Nonetheless we were encouraged by reports of selective STAT3 blockade using small interfering RNA (siRNA) and small hairpin RNAs (shRNA) and by pharmacologic agents such as WP1066, that have inhibited GBM cell proliferation in vitro and tumor growth in vivo [5, 18, 19].

Much remains to be learned about the JAK-STAT pathway as a potential therapeutic target in GBM, and recent data motivated us to pursue the hypothesis that a potent newer generation JAK-STAT targeted inhibitor, SAR31746, could suppress multiple GBM lines representing genetic and phenotypic heterogeneity [18]. Heterogeneity is a hallmark GBM characteristic that has stymied other agents and called into question the use of JAK-STAT inhibition for this disease [18]. Structure based design was used to develop SAR317461 (formerly designated as TG101209; Sanofi-Aventis, Cambridge, MA, USA) and its analog SAR302503, both of which are orally bioavailable and have well-defined pharmacokinetics [20]. SAR302503 is approved for patients in primary myelofibrosis (PMF) and is well tolerated. SAR317461 was designed to be highly JAK2 selective, and it has exhibited marked antitumor activity in several preclinical models [20, 21]. To our knowledge neither compound has ever been tested with GBM cell lines [22, 23].

Here we report initial steps to address the following hypotheses; (1) that SAR317461 can overcome and kill immortalized, serum dependent GBM cell lines, as well as human GBM stem cell (GSC) derived tumorspheres that exhibit the genotypic and phenotypic heterogeneity of the human disease, (2) that the general mechanism of SAR317461 induced GBM cell death is apoptosis, (3) that sensitivity of GBM to SAR317461 depends on the presence of an activated JAK-STAT pathway, and (4) that inhibition of other survival mechanisms, specifically autophagy, may increase the potency of SAR317461. We also sought to determine whether SAR317461 affects the phosphorylation of STAT5 and Akt, in addition to STAT3, as is the case with WP1066 [24]. We present pilot data that for the first time chronicle the effects of SAR317461 on seven patient GSC derived tumorsphere lines and three established, immortalized GBM cell lines. SAR317461 exhibited potent activity against all lines that expressed activated STAT3, and inhibited the phosphorylation of STAT3 but not of STAT5 or Akt. The mechanism of STAT3 induced GBM tumorsphere cell death was via PARP mediated apoptosis, and we further discovered that autophagy inhibitors increased the anti-GBM effect of SAR317461. These novel data which characterize the activity of SAR317461 against a varied array of GBM lines warrant further, more comprehensive explorations of the JAK-STAT pathway as a target for GBM therapy.

## Methods

### TCGA dataset analysis

For Fig. 1 datasets were obtained from the TCGA website (https://tcga-data.nci.nih.gov). The data were derived from 538 adult (18+ years) cases of GBM. This database includes survival information and tumor samples acquired from patients undergo genomic profiling and expression analysis. Tumor samples are required to contain at least 80 % tumor nuclei and no more than 50 % necrosis, and a secondary pathology assessment must confirm the samples represent GBM. For our analysis we used SurvExpress^®^ which is a gene expression database and web-based tool (http://bioinformatica.mty.itesm.mx/SurvExpress) based on several datasets including the TCGA, to provide survival analysis and risk assessment using a biomarker gene list as input to a Cox proportional-hazards regression [25]. Cox regression relates the time of death to a number of explanatory variables known as covariates, in this case genes. For our analysis STAT3 was the specific gene of interest. We analyzed STAT3 gene expression level with respect to GBM in 538 subjects, and survival analysis was censored by survival months, which means that even though patients may have been lost to follow-up, partial data was incorporated in the survival curves. Risk analysis was performed in which a predicted risk for a specific patient genetic profile was determined. The subjects were then partitioned into low risk and high risk groups [25]. STAT3 expression is shown for each risk group (Fig. 1a) and survival was plotted according to the Kaplan–Meier estimator (Fig. 1b). Cox regression and Kaplan–Meier log rank analysis both indicated that the survival data were significant at the 95 % confidence level.

### Reagents for cell based studies

The JAK-2 inhibitor SAR317461 was obtained from Targegen (now Sanofi-Aventis), Alamar Blue was purchased from AbD Serotech, and Anti-pY-STAT3 and anti-STAT3 were purchased from Cell Signaling Inc. Horseradish peroxidase-linked anti-rabbit or mouse IgG were acquired from Jackson ImmunoResearch (West Grove, PA, USA), and Odyssey Inc. supplied Odyssey^®^ Blocking Buffer and IRDye 680.

### Establishment of primary GSC tumorspheres from GBM patients

#### GBM tissue acquisition, processing, and culture of GSCs

We have published on these methods and our tumorsphere lines elsewhere [26, 27]. GBM (grade IV glioma) tumor samples were obtained from 7 adult human (>21 years) surgical patients without the exclusion of either sex or any ethnic/racial groups, under an approved UCSD MCC IRB protocol (IRB #100936), with written, informed patient consent. IRB ethical guidelines were strictly followed, and patient samples were de-identified. The tumors are obtained at surgery and multiple samples are taken covering all parts of the tumor. The tumor samples were immediately washed 2–3 times with 5–10 ml of PBS/NSC basal medium to remove blood and debris, and the tissue was minced for 1–3 min with a No. 10 scalpel blade. The minced tissue was combined and enzymatically dissociated by using 3–5 ml of pre-warmed Accutase^®^ (Life Technologies) for 10–15 min in a 37 °C water bath. The solution was subsequently centrifuged and 10–15 ml of basal medium was added to the tube and filtered through a 40 micron cell strainer to remove clumps and debris. After further washing cells were plated in NSC medium supplemented with 20 ng/ml EGF, 10 ng/ml bFGF and heparin (2 ng/ml), antibiotics added, and the cultures incubated at 37 °C in 5 % CO_2_.

#### Passaging and expansion of patient GBM derived tumorspheres

We cultured five patient derived tumorsphere cell lines GBM4, GBM8, SK1035, SK987, SK892, SK429 and SK262. When the tumorspheres reached an average size of 150–200 μm in diameter, subculture was initiated. The content of each flask was removed and placed in an appropriately sized sterile tissue culture tube, and centrifuged at 190*g* for 6 min at room temperature. The supernatant was removed and the pellet dissociated to create a single cell suspension. The cell suspension was centrifuged, the supernatant was aspirated, and the cells resuspended in 1 ml of NSC medium, and incubated at 37 °C in 5 % CO_2_.

#### Culture of immortalized GBM lines

Human U87, U251 and A172 GBM cells were cultured in Dulbecco’s modified Eagle’s medium (DMEM) supplemented with 10 % fetal bovine serum, 4 mM glutamine, 100 U/ml penicillin and 100 µg/ml streptomycin at 37 °C in 5 % CO_2_–95 % air.

### Cell viability assay

The cytotoxic effect of SAR317461 was determined in triplicate for all 10 GBM lines using the Cell Proliferation Reagent Alamar Blue assay (AbD sciences). Cells (2 × 10^3^ cells/well, 100 µl) added to in 96-well flat-bottomed plates, incubated at 37 °C and 5 % CO_2_–95 % air overnight. After exposure to the JAK2 inhibitor (SAR317461) at concentrations between 0.1 and 40 µM, for 72 h, cell viability was determined by adding Alamar Blue to the cells and 6–12 h later measuring fluorescence using excitation and emission wavelengths of 560 and 590 nm, respectively. Results were expressed as percent viability = [*F1*(treated cells) − background/*F*(untreated cells) − background] × 100 %. Dose–response curves were plotted by using GraphPad^®^ Prism software and EC_50_ values were calculated.

### Western blotting

Untreated and SAR317461 treated human GBM tumorsphere cells were harvested by disassociation, washed with ice-cold PBS, and lysed in NP40 buffer containing 50 mM Tris pH 8.0, 150 mM NaCl, 0.1 % SDS, 1 % NP-40, 0.1 % sodium deoxycholate, protease inhibitor (Complete Mini^®^, Roche Scientific) and Phostop^®^ (phosphotase inhibitor, Roche Scientific). The loading control was GAPDH for all blots. Protein quantification was performed with a BCA protein assay kit from Pierce (Fisher Scientific). Protein was separated by 10 % SDS-PAGE and transferred to nitrocellulose membranes, then transferred to nitrocellulose membranes followed by blocking with Odyssey^®^ Blocking Buffer. Subsequently the membranes were probed with anti-STAT3, antiphospho-STAT3, antiphospho-JAK-2, antiphospho-STAT5 and anti-PARP antibodies (Cell Signaling Inc.), and anti-Bcl-X_L_ (Santa Cruz Biotechnology) and anti-GAPDH antibody (Genetex). After washing with distilled water the membranes were incubated in blocking buffer and IRDye 680 for 1 h under no light conditions, then scanned and read on an Odyssey infrared plate reader (Li-Cor Biosciences).

### Cell cycle analysis

For cell cycle analysis, GBM4 and GBM8 glioblastoma cells were plated at 200,000 cells/well and incubated for 16 h with 2 µM of SAR317461 and DMSO. Cells were dissociated, fixed in ice cold methanol, then incubated at 22 °C in for 30 min PBS containing 50ug/ml RNAse A. Cellular DNA was stained with 250 μl of PI (10 μg/ml), followed by flow cytometric analysis (FACS Calibur^®^ Flow Cytometer, BD Biosciences).

### Autophagy analysis

For the autophagy studies the U251 cell line was engineered to stably express GFP-LC3. 1 µM SAR317461 was added to the medium and then cell viability was measured using the MTT assay. A starvation control was utilized by culturing U251 cells in media without FBS. Also for each category, starvation control, starvation, and JAK2 inhibitor, a vehicle control was also used. In addition to the cell viability assay the cells were imaged on an Olympus fluorescence microscope to assess their morphology.

### Data analysis

For the cell viability studies the overall mean average deviation in percent between replicates used to construct the IC_50_ curves for 10 separate cell lines was calculated to be approximately 8.49 %. The standard deviation of IC_50_ values for seven tumorsphere lines treated with SAR317461 IC_50_ value is approximately 8.1 with a mean of 4.88 (4.88 ± 8.1). Hence the IC_50_ of 25 for SK892, which does not express pSTAT3, is more than 2 standard deviations outside this interval. The IC_50_s for U87, A172 and U251 are 7–8 μM. Statistical differences between cell viability obtained in cells treated with SAR317461, with or without autophagy inhibitors, were determined using a 2-tailed, paired t test and the 95 % level of confidence. Thus test conditions that attained p values of less than 0.05 relative to control were declared to be statistically significant.

## Results

### TCGA data show that STAT3 expression in GBM is associated with lower patient survival

Analysis of TCGA data shown in Fig. 1a depicts STAT3 gene expression according to risk group of GBM patients. The box graph reveals that higher expression of STAT3 is associated with higher risk of GBM. Panel 1b of Fig. 1 indicates that higher expression of STAT3 is associated with lower overall patient survival. Collectively these data suggest that elevated expression of STAT3, which is directly activated by JAK2, is associated with a weaker prognosis in GBM.

### Expression and constitutive activation of STAT3 in glioma cell lines and human tumor neurosphere lines

**Fig. 2:**
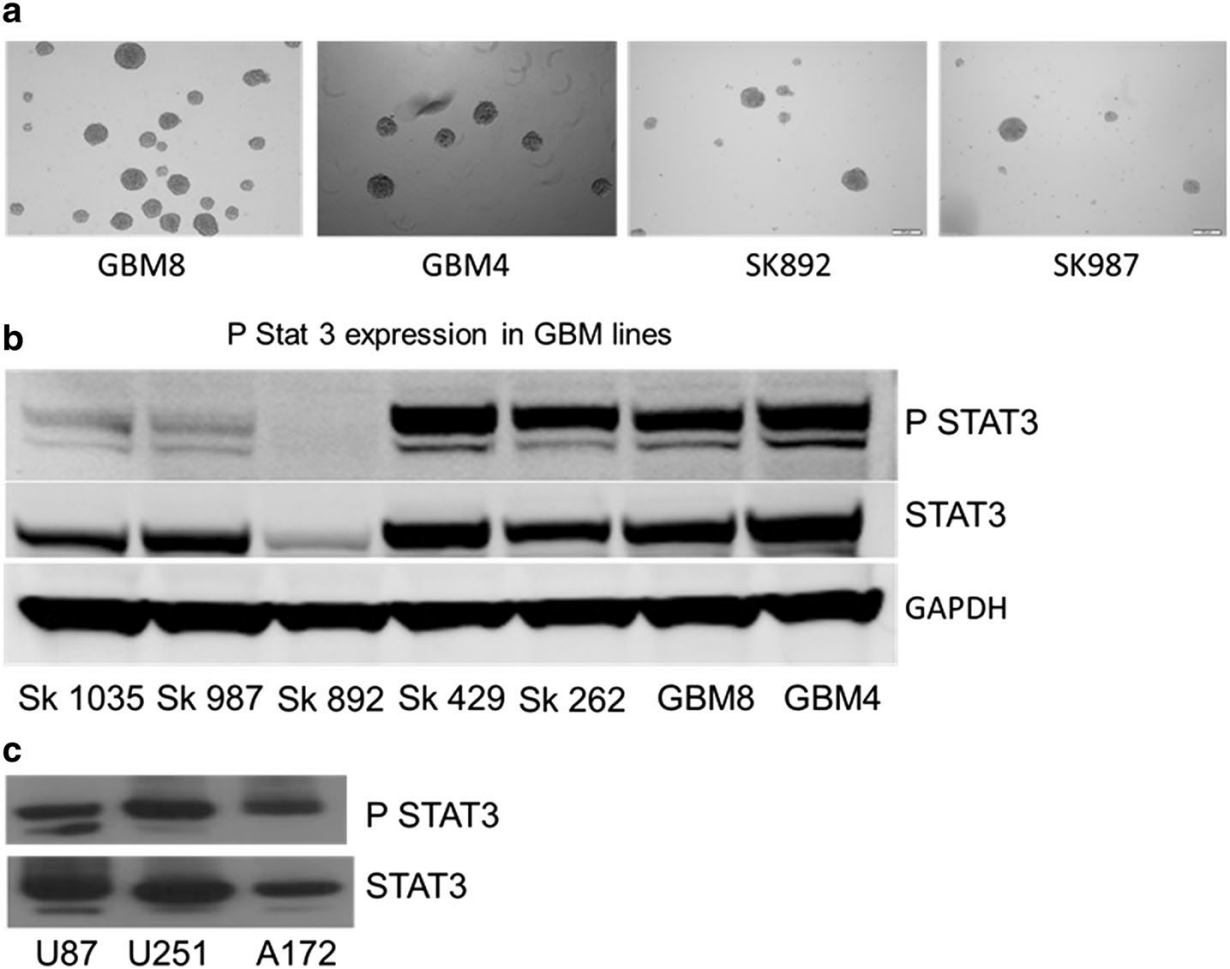
STAT3 phosphorylation status in GBM cell lines. **a** Representative images of untreated tumorspheres derived from GSCs acquired from GBM tumors. The *images* simply illustrate the rounded conformation of healthy tumorspheres. **b** STAT3 phosphorylation in patient derived tumorsphere cell lines. **c** Immortalized adherent cell lines.

Seven GBM-SC lines (GBM4, GBM8, SK262, SK429, SK1035, SK987, SK892) established from GBM patient-derived tumors were successfully grown as neurospheres in neural stem cell medium, as shown by the representative images in Fig. 2a. To determine whether the STAT3 pathway was activated and whether this had any effect on response or non-response to SAR317461, we used immunodetection for phosphorylated STAT3 at Tyr705 in the patient derived GBM lines and in the immortalized A172, U87 and U251 cell lines. Western blot analysis with anti-phosphospecific STAT3 (Tyr705) and anti-STAT3 showed constitutive phosphorylation of STAT3 in all cell lines except SK892 (Fig. 2b, c).

### Cell viability assay

**Fig. 3:**
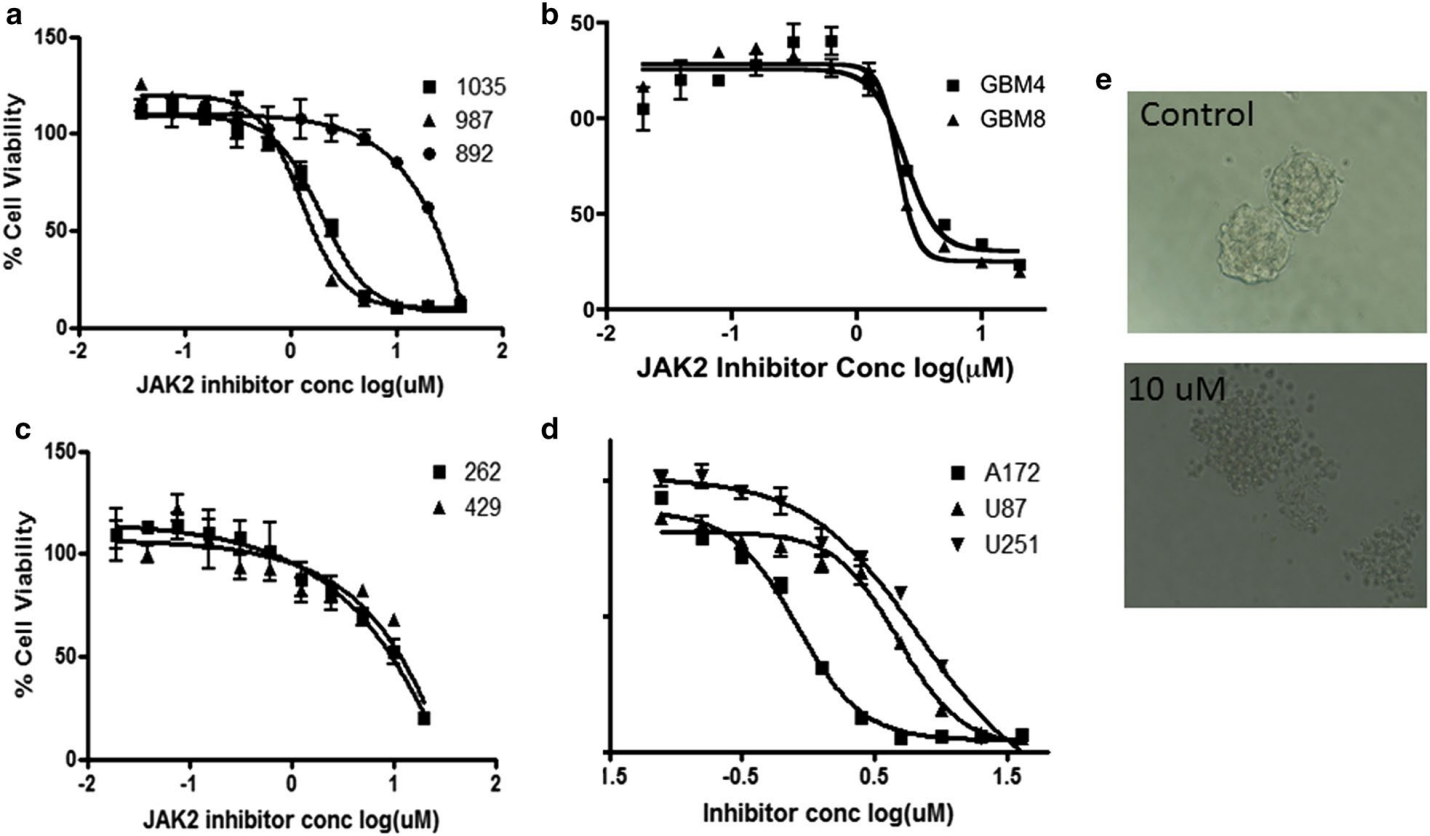
Inhibitory effect of SAR317461 on the proliferation of GBM lines. GSCs and established cells from established GBM lines were seeded into each well of a 96-well micro-culture plate and SAR317461 (40–0.075 μM) was added. *Each point* in *every curve* shows the mean for three samples and the *bars* are standard error of the mean. The standard deviation of IC_50_ values for 7 tumorsphere lines treated with SAR317461 IC_50_ value is approximately 8.1 with a mean of 4.88 (4.88 ± 8.1). Hence the IC_50_ of 25 for SK892, which does not express pSTAT3, lies more than 2 standard deviations outside this interval. The IC_50_s for U87, A172 and U251 are 7-8 μM. **a** (SK1035, SK987 and SK892), **b** (GBM4 and GBM8), **c** (SK262, SK429) are IC_50_ curves for patient derived cell lines. **d** IC_50_ curve of the U87, A172 and U251 immortalized GBM cell lines. **e** Shows representative images of GBM8 cells treated with 10 µM SAR317461 and control solution (DMSO).

We examined the effect of the JAK2 inhibitor SAR317461 on cell proliferation in seven different GBM cell lines in vitro. Treatment with SAR317461 with up to 40 µM of compound for 72 h exhibited a similar inhibitory effect on GBM4, GBM8, SK1035, SK987 stem cells and A172 cell lines with an IC_50_ values of 1–2 µM, whereas in U87 and U251 cell lines the IC_50_ values were between 5 and 8 µM. However, in the GSC derived SK892 tumorsphere line the inhibitory effect was comparatively much lower (IC_50_ ~25 µM) than in the other patient GSC derived lines, possibly because this line did not express pSTAT3 (Fig. 2 & Fig. 3a–e). The mean average deviation in percentage terms between replicates for each cell viability experiment to construct the IC_50_ curves was approximately 8.49 %. The standard deviation of IC_50_ values for 7 tumorsphere lines treated with SAR317461 IC_50_ value is approximately 8.1 with a mean of 4.88 (4.88 ± 8.1). Hence the IC_50_ of 25 for SK892, which does not express pSTAT3, lies more than 2 standard deviations outside this interval. The IC_50_s for U87, A172 and U251 are 7–8 μM. Taken together, these results suggest that SAR317461 can be used to selectively target GBM cells that express activated STAT3 (pSTAT3) (Fig. 3a–e).

### Mechanism of SAR317461 induced cell death in GSC derived GBM tumorspheres

#### Down-regulation of STAT3 phosphorylation

**Fig. 4:**
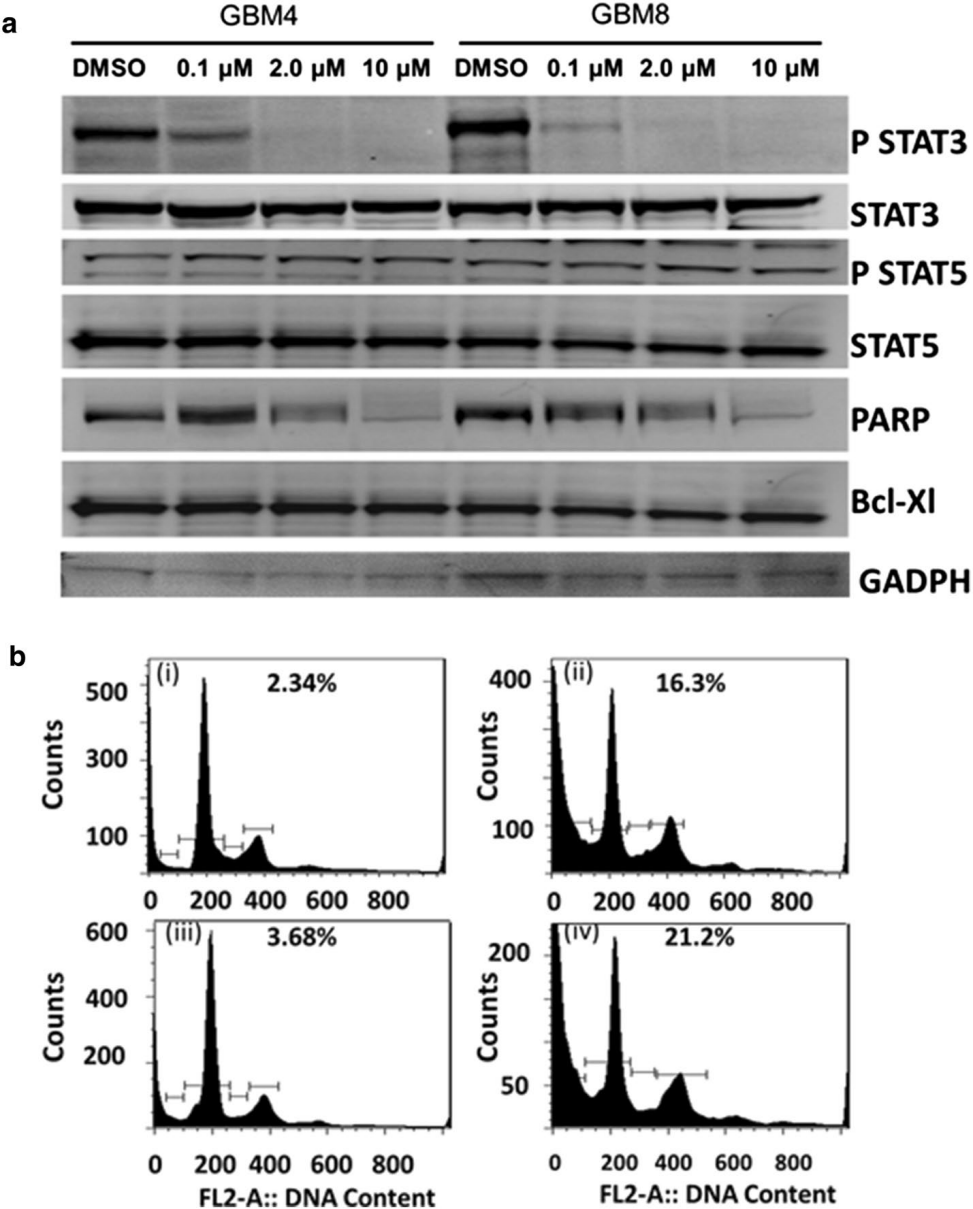
SAR317461 inactivated STAT3, induced PARP cleavage to mediate apoptosis. **a** Western blots of phosphorylated STAT3 at Tyr705 and total STAT3, Bcl-Xl and Parp in GBM4 and GBM8 cells treated with different concentrations of SAR317461 and harvested at 16 h. GADPH loading control. **b** Cells were treated with the 2 µM concentrations of SAR317461 for 16 h and then subjected to flow cytometry for cell cycle analysis. *i* GBM4 cells treated with DMSO. *ii* GBM4 cells treated with 2 uM of SAR317461. *iii* GBM8 cells treated with DMSO. *iv* GBM8 cells treated with 2uM of SAR317461. Percent of cells in sub G1 phase is indicated.

In order to gain mechanistic insight into how the STAT3 pathway is inhibited by SAR317461 in GBM cells, we treated patient GSC derived GBM4 and GBM8 tumorspheres with different concentrations of inhibitor and performed Western Blot analysis for phosphorylated STAT3 levels. In both tumorsphere lines treatment with 10, 2 and 0.1 µM SAR317461 for 16 h resulted in a dose-dependent inhibition of phosphorylation, while 10 and 2 µM SAR317461 rendered STAT3 phosphorylation undetectable (Fig. 4a). STAT5 phosphorylation was not reduced by SAR317461.

#### Induction of PARP cleavage and induction of autophagy

**Fig. 5:**
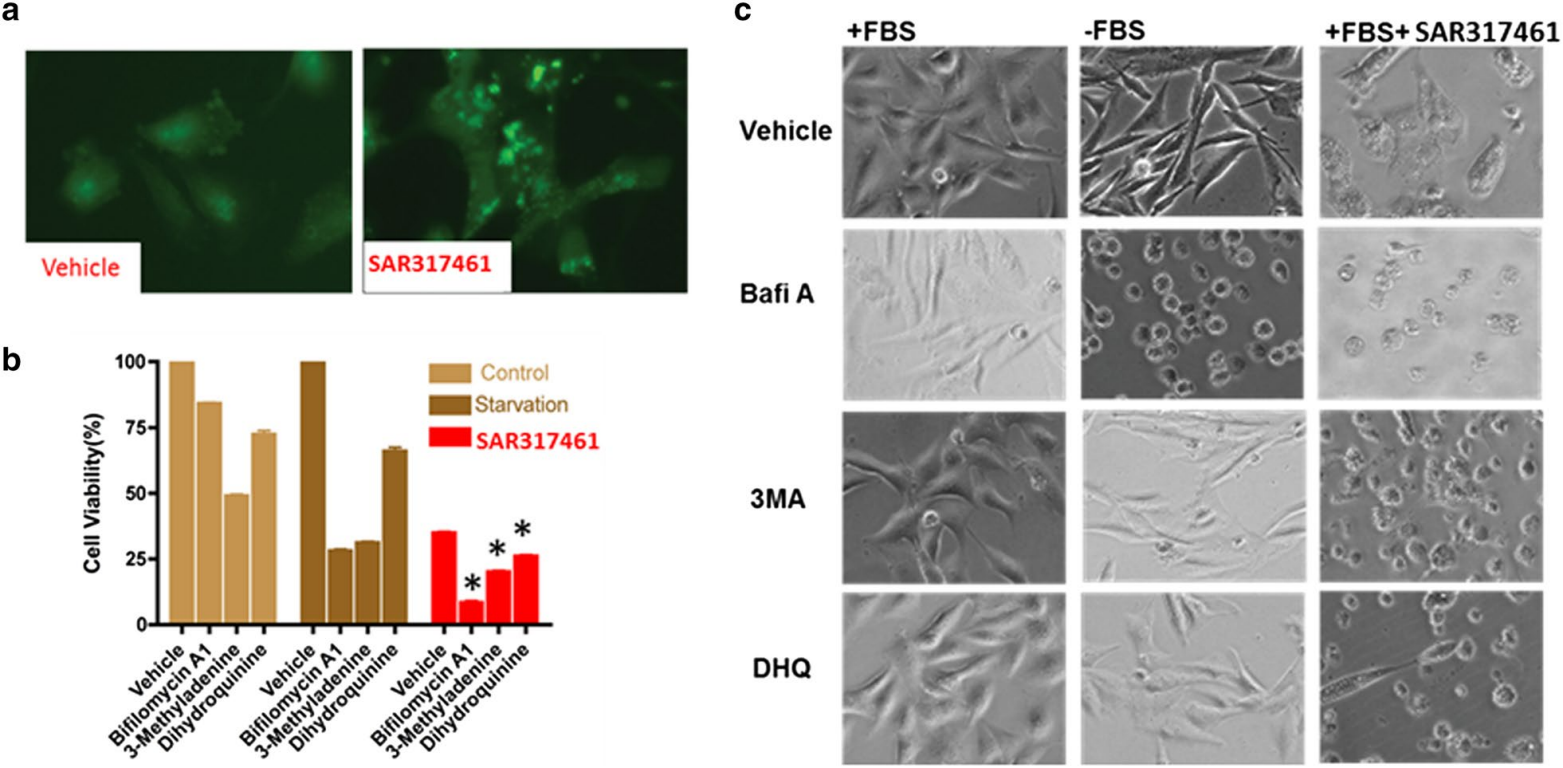
JAK2 inhibitor SAR317461 induced cell autophagy and cell autophagy inhibitors enhanced cell death induced by SAR317461. **a** Stable U251 cell line expressed GFP-LC3 induced GFP clusters with 1 µM SAR317461 treatment. **b** Cell viability decreased dramatically after SAR317461 and cell autophagy inhibitors. There were three replicates per condition and for the starvation control cells were cultured in media without FBS. Statistical differences between cell viability obtained in cells treated with SAR317461 with or without autophagy inhibitors were determined using a 2-tailed, paired t test. *Bars* indicate standard error of the mean and p values of less than 0.05 indicated statistical significance and are denoted by *asterisks* above the *columns*. **c** Photomicrographs showing cell morphology and density with various treatments.

The induction of apoptosis in GBM4 and GBM8 patient derived GSC tumorsphere lines by SAR317461 was evidenced in Western blots by cleaved poly-ADP ribose polymerase (PARP) in the tumorsphere cells (Fig. 4a). The sub-G1 population, which is comprised of apoptotic cells, increased after 16 h of treatment with SAR317461 (Fig. 4b). DNA content analysis by flow cytometry showed increased PI staining of apoptotic cells after treatment with SAR317461 secondary to DNA fragmentation and loss of nuclear DNA content (Fig. 4b). To further elucidate the mechanism of SAR317461 activity we determined whether autophagy occurred after SAR317461 treatment and whether cell death could be enhanced by inhibiting autophagy, which has a protective role. We found that SAR317461 induced cell autophagy and autophagy inhibitors enhanced cell death induced by SAR317461 (Fig. 5a–c).

## Discussion

STAT3 is a key intracellular effector for cytokine and growth factor-dependent transcriptional activation of genes that control proliferation, differentiation and apoptosis of a wide range of normal as well as transformed cell types [7]. Moreover, recent studies have indicated that STAT3 expression in GBM is elevated (Fig. 1), and that GBM tumors and cell lines exhibit high levels of constitutively activated STAT3 relative to normal human astrocytes, white matter, and normal tissue surrounding GBM tumors [9]. However, in preclinical models STAT3 has been reported to play either an oncogenic or suppressive role depending on the genetic background of the tumor system, and it is this variability that has in part complicated the preclinical development of JAK-STAT inhibitors [17]. For example de la Iglesia et al. [28] found that when EGFR is mutated STAT3 is oncogenic, but in tumors with PTEN mutations STAT3 can act as a tumor suppressor. The basis for this apparent functional difference is unknown. Moreover, preclinical results with previous JAK-STAT inhibitors produced variable levels of anti-GBM activity [22]. However the present study demonstrated that inhibition of STAT3 activation with the potent JAK2 inhibitor SAR317461 effectively induced apoptosis in a heterogeneous array of human GSC derived tumorspheres and established GBM cell lines. SAR317461 also triggered autophagy and importantly, the addition of an autophagy inhibitor led to markedly enhanced cell killing.

A major difficulty in targeting GBM is the heterogeneity of mutations and signaling aberrations that occur within a single tumor and between patient tumors [29]. The intra- and extratumoral heterogeneity of multiple GBM tumorsphere lines we tested did not appear to prevent SAR317461 from exerting potent anti-tumor activity. SAR317461 suppressed cell viability in several patient derived GSC lines with an IC_50_ of 1–2 µM, as well as established GBM lines with an IC_50_ of 2–10 µM (Fig. 3). Notably in one patient derived line, SK892, which had no constitutive expression of activated STAT3 (pSTAT3) the IC_50_ was much higher (25 µM) than the other GSC lines. This finding is isolated but does align with the possibility that pSTAT3 expression may be associated with the ability of SAR317461 to exert a comparatively potent suppressive effect on GBM tumors.

The present study suggests that the mechanism by which SAR317461 inhibits GBM tumorspheres is apoptosis and that PARP cleavage plays a role. The relevant pathways remain to be identified, although it is known that targets of STAT3 include various prosurvival genes including those for Bcl-2, Mcl-1, and Bcl-X_L_ [17]. Furthermore, it has been reported for multiple myeloma that SAR317461 induced down regulation of pJAK2 and pSTAT3 levels that correlated with up-regulation of pErk and pAkt, indicating cross talk between these signaling pathways that is yet to be delineated in detail [22]. Down regulation of the antiapoptotic protein Bcl-X_L_ has been reported in myeloma and U251 cells with JAK2 inhibition, but curiously, in our patient derived GSC tumorspheres down-regulation of Bcl-X_L_ was not apparent in the Western blot analysis we performed [9, 30]. The reasons for this are unclear, although it is possible that SAR317461 induction of PARP cleavage and apoptosis in GSC tumorspheres is based on different signaling than in multiple myeloma and immortalized GBM lines. For example Epling-Burnette et al. [31] reported that induction of apoptosis was independent of Bcl-X_L_ regulation in large granular lymphocyte (LGL) leukemia. Instead, they found reduced protein expression of another Bcl-2-family protein, Mcl-1 which is known to be modulated by STAT3 [13].

Interestingly we also found that SAR317461 induced autophagy in tumorspheres. Autophagy can have a cytoprotective role in tumor cells and we found that cell killing was markedly enhanced by the addition of the autophagy inhibitor bifilomycin A, suggesting an opportunity for combined therapy. SAR317461 was designed to be JAK2 selective, and it would of interest to learn in the context of drug resistance, whether it affects the same targets as WP-1066, which is a JAK-STAT inhibitor that has shown promise and which may soon enter clinical trials for GBM. WP1066 has been found to variously impact multiple intracellular targets, including the inhibition of phosphorylation of STAT3 and STAT5 and AKT, and activation of immune effector cells [24, 32]. SAR317461 which was intended to be JAK2 selective, did not inhibit STAT5 and Akt phosphorylation, suggesting a differences in activity profiles between WP1066 and SAR317461 even though both induce apoptosis [18].

SAR317461 may be useful for disseminated GBM and may have broader relevance beyond GBM for several reasons. STAT3 impacts stem cell maintenance in general as the interaction between STAT3 and other pathways including EGFR, Notch, Wnt, Hedgehog, Akt, mTOR, olig2, PKC, MAPK, NF-κB and BMP4 has been shown to regulate self-renewal [33]. Hence an advantage of targeting STAT3 in GBM is that the inhibition of this pathway affects multiple downstream molecules in the GSC tumor compartment which drives GBM and is invasive [34]. In terms of other central nervous system (CNS) diseases, it is perhaps important that STAT3 plays a role in astrocyte differentiation and activity, and moreover astrocytes are immunocompetent cells and are capable of secreting inflammatory mediators that may be involved in CNS pathology such as Alzheimer’s disease, multiple sclerosis, Parkinson’s disease and brain injury [35, 36]. So, SAR317461 could potentially be therapeutically active in CNS diseases involving dysfunction of astrocytes or astrocyte precursors.

## Summary and conclusions

For this pilot study a limited number of GBM lines were used although all possible steps were taken to ensure that the data were internally consistent. Cell based studies utilized the same experimental methodology and analysis approach. The overall mean average deviation in percent between replicates used to construct the IC_50_ curves for 10 separate cell lines was calculated to be less than 10 % (8.49 %). For Western blot analysis a loading control was used to ensure that the intensity of the visualized bands was real and not an artifact of different loaded concentrations of the protein of interest. For studies in which the effect of combining autophagy inhibitors with SAR317461 was evaluated, we utilized both starvation controls and control solutions (PBS).

Our data demonstrate that SAR317461 potently inhibited both STAT3 phosphorylation and induced apoptosis in multiple primary patient derived GSC based GBM tumorspheres which are genetically and phenotypically heterogeneous both internally and with respect to each other. SAR317461 also exhibited significant activity against three immortalized serum-dependent human GBM cell lines. One patient derived GBM line that did not express activated STAT3 showed a comparatively much reduced response to SAR317461, suggesting that pSTAT3 may need to be present for potent SAR317461 associated anti-GBM activity, a finding that could have relevance from a personalized treatment perspective. Future explorations with a wider range of GBM lines variously expressing activated STAT3 would bypass the size limitation of the present study and may more precisely define the possible dependence of SAR317461 GBM response on the presence of activated STAT3. Moreover, patient derived tumorsphere lines and other patient sourced cancer cell types resistant to standard therapy should be explored with SAR317461 both singly and with other agents and pathway inhibitors. Follow-on mechanistic studies in all lines may identify a possible role of other Bcl-2 family proteins in the context of SAR317461 induced apoptosis. Finally, orthotopic GBM models may help characterize tumor and brain concentrations of SAR317461 and its effects alone and in combination on GBM tumor proliferation, invasion, and angiogenesis. On the basis of the pilot results reported herein for SAR317461, we believe that the JAK-STAT pathway does merit further and comprehensive testing as a potential therapeutic target for GBM.

## Abbreviations

CNS: central nervous system
GBM: glioblastoma
GSC: Glioblastoma Stem Cell
JAK2: Janus kinase 2
nM: nanoMolar
STAT: signal transducer and activator of transcription
TCGA: The Cancer Genome Atlas
TMZ: temozolomide
μM: microMolar
μm: micrometer
